# In vitro ruminal fermentation, methane production and nutrient degradability as affected by fruit and vegetable pomaces in differing concentrations

**DOI:** 10.1111/jpn.13656

**Published:** 2021-10-26

**Authors:** Katrin Giller, Laura Bossut, Lukas Eggerschwiler, Melissa Terranova

**Affiliations:** ^1^ Department of Environmental Systems Science ETH Zurich Zurich Switzerland; ^2^ Agroscope Posieux Posieux Switzerland; ^3^ Present address: ONIRIS Nantes‐Atlantic National College of Veterinary Medicine, Food Science and Engineering Nantes France

**Keywords:** aronia, methane mitigation potential, polyphenol, pomegranate, short‐chain fatty acid, tannin

## Abstract

Pomaces are food industry by‐products and may serve as animal feed to increase sustainability of meat and milk production. The aim of the present study was to evaluate fermentation characteristics of dried fruit and vegetable pomaces in a short‐term in vitro experiment using the Hohenheim Gas Test. A selection of six fruit (apple, aronia, orange, pomegranate, red, white grape) and three vegetable (beetroot, carrot, tomato) pomaces was tested in three concentrations (150, 300, 500 g kg^−1^ of dry matter (DM)) as supplement to the basal diet (hay, used as control). Three runs were performed, using rumen fluid from one of three different rumen‐cannulated cows in each run. Per run, each compound was tested in duplicate. After 24 h incubation, total gas production, methane and CO_2_ concentration, short‐chain fatty acids, in vitro organic matter digestibility as well as microbial counts were determined. In addition, the pomaces' polyphenol content including the fractions non‐tannin phenols, condensed tannins and hydrolysable tannins were analysed. Most pomaces did not significantly affect rumen fermentation characteristics in any of the tested dosages and may thus be applied in ruminant nutrition without adverse effects. Aronia significantly decreased (−14.5%) the organic matter digestibility in the highest concentration whereas apple (+12%), carrot (+10%) and beetroot (+8%) increased gas formation related to digestible organic matter. The 500 g kg^−1^ dosage of pomegranate significantly decreased methane formation by about 28% without impairing digestibility. Pomegranate was the only pomace of those high in total tannins that contained exceptionally high amounts of hydrolysable (90% of total tannins) and proportionally low amounts of condensed tannins (10% of total tannins), indicating that the hydrolysable tannins most likely reduced the methane production. Therefore, pomegranate pomace may be an interesting option for a methane mitigating feed supplement in ruminants and should be considered for following in vivo testing.

## 1 INTRODUCTION

The demand for a more sustainable production of animal‐derived foods increases the interest in utilizing food industry by‐products in livestock nutrition. In this respect, a potentially interesting food industry by‐product is pomace, derived from fruits and vegetables processing. Pomace is the pressing residue that remains after juice, wine and cider production. It consists of seeds, stems, peel and pulp in varying proportions. Worldwide, millions of tons of pomace are produced annually and need to be disposed of (Fuentes et al., 2004; Shalini & Gupta, 2010). This disposal is problematic for various reasons. First, adequate disposal of pomaces is quite costly with an estimated 10 million US dollar spent annually in the US to dispose of pomace produced solely from apple (Shalini & Gupta, 2010). The high amounts of sugars, fibre, protein, lipids and bioactive components present in pomaces lead to environmental pollution when pomaces are disposed on landfills, while not using these nutrients also is a waste of resources (Osorio et al., 2021). Therefore, pomaces are partly processed in secondary applications such as extraction of pectin and bioactive compounds for supplements in human nutrition, production of spirits and utilization as fertilizer (Shalini & Gupta, 2010; Varzakas et al., 2016). Furthermore, pomaces are used for energy production in biogas reactors (Shalini & Gupta, 2010). A small portion of these valuable by‐products is re‐introduced into the food chain through the utilization as livestock feed. The high crude fibre content makes pomaces an interesting component for ruminant nutrition. Some pomaces, for example those derived from apple or carrot, can also be rich in sugars (Bao & Chang, 1994; Sato et al., 2010) and might thus negatively affect rumen fermentation and increase methane production (Ellis et al., 2012).

Only few studies have investigated the effects of pomace feeding on feed efficiency, productivity, product quality, greenhouse gas emission, nitrogen excretion and ruminant health. The replacement of maize and barley by grape pomace in a dairy cow ration did not affect milk yield whereas feeding Indian gooseberry pomace to lactating buffaloes increased milk yield and milk production efficiency (Chedea et al., 2017; Singla et al., 2021). These examples indicate that feeding pomaces to ruminants may maintain or even increase productivity. Feeding ensiled grape pomace to dairy cows increased the proportion of polyunsaturated fatty acids (PUFA) in milk fat whereas milk PUFA proportions were not affected by intake of Indian gooseberry pomace by buffaloes (Santos et al., 2014; Singla et al., 2021). Since pomaces, especially the ones derived from dark red fruits, contain large amounts of polyphenols such as tannins, they may potentially mitigate methane emissions (Naumann et al., 2015) and ammonia excretion, thereby decreasing liver stress and environmental pollution (Patra & Saxena, 2011). Increased plasma polyphenol concentrations were observed in dairy cows after grape pomace intake (Chedea et al., 2017). Feeding aronia pomace to lambs increased the antioxidant properties of plasma and liver tissue (Lipińska et al., 2017). Inclusion of fruit and vegetable wastes in sheep resulted in reduced methane and nitrous oxide emissions while plasma total antioxidant capacity was enhanced (Sahoo et al., 2021). Therefore, beneficial effects of pomace intake on animal health, product quality and sustainability of animal‐derived food production could be assumed.

The pomaces predominantly investigated in vivo as feedstuff for ruminants are apple, citrus fruits, grape and tomato pomace (Abbeddou et al., 2011; Bakshi et al., 2016; Chedea et al., 2017; Wadhwa et al., 2015). However, the juice industry processes a much larger variety of fruits and vegetables and consequently, the variety of the resulting pomaces is large as well but their fermentation characteristics including methane mitigation potential are poorly investigated.

We hypothesized that: (a) Low proportions of pomaces (150 g kg^−1^) can be applied without large changes in rumen fermentation parameters whereas high pomace proportions (500 g kg^−1^) impair the fermentation processes. (b) In vitro organic matter digestibility (IVOMD) decreases with increasing polyphenol content of the pomaces; (c) Pomaces with a high tannin content reduce total gas and methane formation compared to hay and pomaces with a low tannin content.

For this purpose, the present study used the Hohenheim Gas Test to evaluate the applicability of fruit and vegetable pomaces (precisely pomaces of apple (*Malus domestica*), aronia (*Aronia melanocarpa*), orange (*Citrus sinensis*), pomegranate (*Punica grantum*), red and white grape (*Vitis vinifera*), beetroot (*Beta vulgaris*), carrot (*Daucus carota*) and tomato (*Solanum lycopersicum*)) in three concentrations (150, 300 and 500 g kg^−1^ of basal diet), for ruminant nutrition. Specifically, the effect of the treatments on several fermentation characteristics and potential side‐effects on nutrient degradability and methane formation in vitro were investigated. In addition, the classes of polyphenols (non‐tannin polyphenols, condensed tannins, hydrolysable tannins) present in the pomaces were characterized.

## 2 MATERIALS AND METHODS

Rumen fluid for the in vitro incubations was collected from rumen‐cannulated cows according to approval number ZH 38/14 and approval number 2016_11_FR of the respective Cantonal Veterinary Offices in Switzerland.

### 2.1 Preparation of pomaces

Dried aronia pomace (AR) was obtained from IG Aronia, Switzerland. All other fruits and vegetables were purchased during June 2018 at local groceries stores and underwent standardized processing into pomace to avoid introducing a bias by differing processing of the raw material (apples [AP; Gala, class 1, furnished by Tobi Seeobst AG, B'zell; purchased from Migros), oranges (OR; class 1, variety Valencia late, calibre 70–80 mm, furnished by Rodi Fructus AG, 4313 Möhlin, produced by Albenfruit, S.L.U. ES‐46680 Algemesi; purchased from Migros), pomegranates (PG; purchased from Coop), white (WG; Vittoria; purchased from Migros) and red (RG; Black Magic; purchased from Migros) grapes, beetroots (BR; purchased from Migros), carrots (CA; furnished by Minog AG 9445 Rebstein, produced by Müller azmoos ag 9476 Weite) and tomatoes (TO; « Aus der Region», Migros)). Each type of fruit and vegetable was juiced separately (PJ 850, Primotecq, Power max. 850 W, Fust AG, Oberbüren, Switzerland). The resulting pomace was collected. The recovery of pomace varied from 9 (red grape) to 57% (pomegranate). For oranges and pomegranate fruits, the peel of the fruits was chopped and mixed with the respective pomace. Pomaces were dried in an oven at 60°C for 72 h. The dried pomaces as well as grass hay (ryegrass‐dominated) as control diet were ground to pass through a 1 mm screen with a centrifugal mill (Model ZM1; Retsch GmbH).

### 2.2 In vitro incubation

In vitro incubations of dried pomaces were performed using the Hohenheim Gas Test (HGT) method according to Menke and Steingass (1988). Rumen fluid for the individual runs was collected from three rumen‐cannulated cows before the morning feeding (approval number ZH 38/14: cow 1, Tierspital Zurich; approval number 2016_11_FR: cows 2 and 3, Agroscope Posieux) on two different days per cow. The pH and bacterial counts of all rumen fluids were determined before incubation. The six batches of rumen fluid used in the experiment had a pH range 6.1–7.1 and 1.5–2.3 × 10^9^ bacteria/ml rumen fluid. Cows were kept according to the Swiss guidelines for animal welfare. Cow 1 was fed a total mixed ration (TMR) whereas cows 2 and 3 had access to pasture all day and night and received an additional 8–10 kg (fresh matter) of maize silage per day. After collection, the rumen fluid was filled into thermos flasks until overflowing and then closed without any remaining air inside before being transport to the lab. To remove larger feed particles before incubation, the rumen fluid was passed through a four‐layered gauze (1 mm pore size). One part of the filtered rumen fluid was mixed with two parts of preheated (39°C) reduced buffer solution (prepared according to Menke & Steingass, 1988) that was continuously sparged with carbon dioxide. Scaled glass syringes with two outlets (Soliva & Hess, 2007) were prepared with 200 mg dry matter (DM) of basal diet alone (grass hay, without pomace) or with one of the nine dried pomaces in amounts of 150, 300 and 500 g kg^−1^ of total DM, replacing the respective proportion of grass hay in the syringe. The chemical compositions of hay and dried pomaces are presented in Table 1. Each treatment (*n* = 28) was included in duplicate in at least three out of the six runs. Individual rumen fluid from each of the three rumen‐cannulated cows was used at least once per treatment duplicate (*n* = 6 per pomace, *n* = 12 for basal diet (without pomace)). Thirty ml of buffered rumen fluid were filled into each prepared glass syringe followed by anaerobic incubation at 39°C and continuous rotation for 24 h (Soliva & Hess, 2007). For each run, two syringes containing only rumen fluid without any feed added served as a blank. After 24 h of incubation, the produced total gas volumes were recorded. Subsequently, the buffered rumen fluid was removed from the syringes and transferred to collection tubes to terminate the fermentation and keep gas and liquid separately for subsequent analyses.

**Table 1:** Chemical composition of the incubated components (g kg^−1^ dry matter).

	hay	AP	AR	BR	CA	OR	PG	RG	TO	WG
Dry matter (DM)^a^	956	973	930	969	962	946	948	957	953	954
Organic matter	933	982	977	931	934	963	959	965	932	918
Crude protein	160	77.8	64.5	22.6	85.8	73.3	130	65.3	60.4	60.9
aNDFom	641	306	389	377	907	222	302	353	522	272
ADFom	411	244	330	216	218	181	237	325	428	264
Lignin(sa)	114	121	226	102	97.6	59.1	118	240	132	156
Ether extract	20.4	18.8	39.7	2.48	NA	19.6	33.1	15.2	78.8	15.7
Polyphenols (g kg^−1^ DM)										
TEP	12.7	3.87	107	4.63	2.17	18.6	115	58.6	2.87	32.6
NTP	8.33	1.07	12.2	3.64	1.49	15.2	4.59	3.13	2.75	1.86
TT	4.35	2.80	94.8	0.99	0.68	3.5	111	55.5	0.12	30.7
CT	0.48	1.39	79.6	0	0.18	0.4	10.3	54.0	0.08	30.5
HT	3.86	1.41	15.2	0.99	0.05	3.1	100	1.42	0.04	0.18

Abbreviations: ADFom, acid detergent fibre after ashing; aNDFom, neutral detergent fibre after amylase treatment and ashing; AP, apple; AR, aronia; BR, beetroot; CA, carrot; CT, condensed tannins; HT, hydrolysable tannins; NA, not analysed; NTP, non‐tannin phenols; OR, orange; PG, pomegranate; RG, red grape; TEP, total extractable phenols; TO, tomato; TT, total tannins; WG: white grape.

^a^ In g kg^−1^ original substance.

### 2.3 Chemical composition of basal diet and pomaces

For the hay (basal diet) as well as the dried pomaces, DM, total ash (TGA 701; Leco Corporation; AOAC index no. 942.05; AOAC (AOAC, 1997)), nitrogen (N) (TruMac CN; Leco Corporation; AOAC index no. 968.06), aNDFom (Fibretherm FT 12 (Gerhardt) according to Van Soest et al. (1991) with the addition of heat‐stable amylase, without sodium sulfite), and ether extract (extraction System B‐811; Büchi; AOAC index no. 963.15) were analysed.

### 2.4 Analyses of fermentation parameters

The concentrations of methane and CO_2_ in the total fermentation gas were determined by gas chromatography (6890 N; Agilent Technologies; calibration gas from PanGas) equipped with a thermal conductivity detector (Soliva & Hess, 2007). The pH in the incubated buffered rumen fluid was determined using a digital pH meter (Metrohm). Bacteria and protozoa were fixed in Hayem solution (HgCl_2_ 9 mmol/L, Na_2_SO_4_ 176 mmol/L, NaCl 86 mmol/L) and diluted formaldehyde (0.04 mmol/L w/v in water), respectively. Bürker counting chambers (Blau Brand) with a depth of 0.02 mm for bacteria and 0.1 mm for protozoa were used to determine microbe numbers. The analysis of short‐chain fatty acids (SCFA) in the incubation fluid was performed by high‐performance liquid chromatography (HPLC) (LaChrom, L‐7000 series, Hitachi Ltd., Japan) following Ehrlich et al. (1981).

### 2.5 Quantification of polyphenols in basal diet and pomaces

Polyphenols were extracted from hay and pomaces using 70% acetone. The total extractable phenol (TEP) and non‐tannin phenol (NTP) contents in the pomaces were analysed using the Folin‐Ciocalteu colorimetric method (Jayanegara et al., 2011). The determination of condensed tannins (CT) was performed with the butanol‐HCl‐iron method (Makkar, 2003) and the contents were given as leucocyanidin equivalents. The total tannin (TT) and hydrolysable tannin (HT) contents were calculated from the analysed results.

### 2.6 Calculations and statistical analysis

To obtain the net gas production, the total gas production from the blank was subtracted from total gas production from test samples. Calculation of the *in vitro* organic matter digestibility (IVOMD) was performed according to the standard equation of Menke and Steingass (1988) as IVOMD (g kg^−1^) = 148.8 + 8.893 × total gas production (ml 200 mg^−1^ DM) + 0.448 × crude protein (g kg^−1^ DM) + 0.651 × ash (g kg^−1^ DM).

Six in vitro runs using individual rumen fluid from three different cows were performed in total. Due to limited capacity in the rotor used for the incubation, two sets of test compounds (set 1: basal diet, AP, AR, OR, RG, WG in all three concentrations; set 2: basal diet, BR, CA, PG, TO in all three concentrations) were incubated in three runs each, using rumen fluid from each cow once per set. Treatments were incubated in duplicate per run, leading to six values per treatment to be included in the statistical analysis. Data were analysed using a mixed model in SPSS (version 26, IBM) with type of pomace, dosage and their interaction as fixed factors and donor cow and run as random factors. Multiple comparisons were performed using the Games‐Howell post hoc test. Statistical significance was considered at *p* ≤ 0.05 and a trend at 0.05 < *p* ≤ 0.1.

## 3 RESULTS

### 3.1 Composition of the dried pomaces

The DM content of the dried pomaces varied between 930 and 973 g kg^−1^ (Table 1). The hay used as the basal diet had an intermediate DM content of 956 g kg^−1^. The organic matter content was highest in AP and AR and lowest in WG. The PG had a crude protein (CP) content rather close to that of hay (−19%) whereas the CP contents of all other pomaces were only about 50% or less of that of hay (minimum: BR with −86% compared to hay). The ADFom content of TO was comparable to that of hay and minimum ADFom contents were observed for BR and CA (about −50% compared to hay). In AR and PG, the lignin(sa) content was almost doubled compared to hay whereas it was especially low in OR (−50% compared to hay). The ether extract content was 4‐ and 2‐fold higher in TO and AR compared to hay. In BR, ether extract content was only about 10% of that found in hay and all other pomaces.

**Figure 1:**
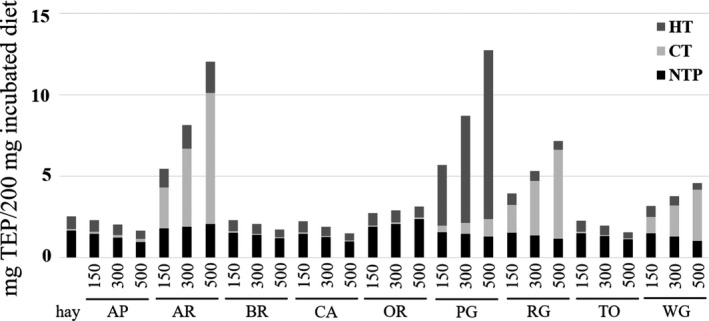
Calculated amounts of total extractable phenols (TEP), classified as hydrolysable tannins (HT), condensed tannins (CT) and non‐tannin phenols (NTP), in syringes at the start of incubation. AP, apple; AR, aronia; BR, beetroot; CA, carrot; OR, orange; PG, pomegranate; RG, red grape; TO, tomato; WG, white grape

The higher TEP content in hay compared to AP, BR, CA and TO (Table 1) resulted in a higher amount of TEP in the incubation syringe containing only hay compared to all combinations of hay with one of those four pomaces (Figure 1). As AR, OR, PG, RG and WG had higher TEP concentrations than hay (Table 1), TEP concentration in the syringes increased with increasing pomace concentration. While the TEP in syringes with only hay as well as with all concentrations of AP, BR, CA, OR and TO were composed of about 57–75% NTP, 22–32% HT and at maximum 12% of CT, the TEP in syringes with all concentrations of AR, RG and WG contained a much larger proportion (33–76% of TEP) of CT (Figure 1). In contrast, the majority of the TEP in the PG syringes were HT (66%–82% of TEP).

### 3.2 In vitro fermentation characteristics

In the following, the concentrations of the pomaces tested are abbreviated with 150, 300 and 500 for the concentrations 150, 300 and 500 g kg^−1^, respectively. Total gas production per g DM during 24 h was significantly increased compared to the respective hay control with AP500 and CA500 by 29 and 22%, respectively, whereas it was significantly decreased by 15% with AR500 (Table 2). When calculated in relation to digestible organic matter (dOM), AP300, AP500, BR500, CA500 and WG500 significantly increased gas production during 24 h compared to the respective hay control. All other dosages of AP, CA and AR as well as all other pomaces at any dosage did not affect the total gas production per g DM or per g dOM. Concomitant to the total gas production per g DM, the in vitro organic matter digestibility (IVOMD) as well as the dOM were significantly increased by AP500 and significantly decreased by AR500 compared to only hay. The CA500 significantly increased methane formation per g DM by 17% compared to only hay. Contrarily, PG500 significantly decreased methane formation per g DM as well as methane formation per g dOM (−28% and −20%). All other pomaces did not significantly affect methane production. The ratio of methane to CO_2_ formation and the CO_2_ formation per g DM were not affected by any of the pomaces in any dosage compared to the basal diet.

**Table 2:** Effects of different fruit and vegetable pomace concentrations (conc.) on in vitro rumen fermentation characteristics at dosages of 150, 300 and 500 g kg^−1^ of basal diet in comparison to a non‐supplemented control (*n *= 6)

	Dosage g kg^−1^	AP	AR	BR	CA	OR	PG	RG	TO	WG	SEM	*p*‐values		
												pomace	conc.	*p* × c
Total gas/DM (ml g^−1^)	0	217^Y^	217^Y^	229	229^Y^	217	229	217	229	217^YZ^	6.90	<0.001	<0.001	<0.001
	150	232^Y^	206^YZ^	229	235^YZ^	222	203	200	228	218^Y^				
	300	250^bcYZ^	200^aYZ^	236^abc^	253^cYZ^	225^abc^	198^ab^	206^a^	231^abc^	215^abYZ^				
	500	281^eZ^	184^aZ^	253^de^	280^eZ^	242^bcde^	193^abc^	204^ab^	232^abcde^	238^cdZ^				
IVOMD (g kg^−1^)	0	649^Y^	649^Y^	602	602	649	602	649	602	649	2.17	<0.001	0.998	0.196
	150	665^YZ^	619^Y^	661	676	649	619	609	662	645				
	300	686^cdYZ^	597^aYZ^	664^bcd^	702^d^	646^abcd^	606^ab^	611^ab^	660^bcd^	634^abc^				
	500	728^dZ^	555^aZ^	607^abcd^	743^d^	664^bcd^	590^abc^	594^ab^	653^abcd^	668^c^				
dOM (mg day^−1^)	0	121^Y^	121^Y^	112	112	121	112	121	112	121	4.04	<0.001	0.925	0.177
	150	125^Y^	116^Y^	123	126	122	116	114	124	120				
	300	130^bcYZ^	113^aYZ^	124^abc^	131^c^	122^abc^	114^a^	115^a^	123^abc^	118^ab^				
	500	139^dZ^	106^aZ^	113^abcd^	139^d^	126^bcd^	112^abc^	113^ab^	122^abcd^	124^c^				
CH_4_/DM (ml g^−1^)	0	34.7	34.7	36.4	36.4^Y^	34.7	36.4^Y^	34.7	36.4	34.7	2.60	<0.001	0.380	<0.001
	150	35.8	32.8	36.2	34.4^YZ^	36.2	31.5^Z^	32.2	35.5	35.1				
	300	36.4	27.4	39.0	39.8^YZ^	34.3	31.5^YZ^	33.6	38.6	35.7				
	500	43.6^abc^	28.0^ab^	40.9^bc^	42.6^cZ^	37.5^abc^	26.1^aZ^	30.6^abc^	37.1^bc^	32.3^abc^				
CO_2_/DM (ml g^−1^)	0	180	180	195	195	180	195	180	195	180	13.1	<0.001	0.004	0.184
	150	187	173	178	179	167	139	159	172	180				
	300	191	149	188	218	175	165	172	172	180				
	500	230^c^	155^a^	189^abc^	209^abc^	199^abc^	155^ab^	171^abc^	202^bc^	182^abc^				
Total gas/dOM (ml g^−1^)	0	359^X^	359	367^Y^	367^Y^	359	367	359	367	359^Y^	4.53	<0.001	<0.001	<0.001
	150	371^XY^	355	372^Y^	373^Y^	365	350	350	370	363^Y^				
	300	384^cYZ^	354^a^	381^bcYZ^	386^cYZ^	369^abc^	347^a^	358^ab^	375^bc^	365^abcYZ^				
	500	403^dZ^	348^a^	397^cdZ^	403^dZ^	384^abcd^	344^ab^	362^a^	380^abcd^	385^bcZ^				
CH_4_/dOM (ml g^−1^)	0	57.3	57.3	58.2	58.2	57.3	58.2^Y^	57.3	58.2	57.3	4.03	0.001	0.678	0.007
	150	57.1	56.2	58.6	54.6	59.8	54.4^Y^	56.3	57.5	58.5				
	300	55.9	48.4	62.9	60.6	56.6	55.2^YZ^	58.6	62.9	60.3				
	500	62.4^ab^	52.8^ab^	64.1^b^	61.4^b^	59.7^ab^	46.7^aZ^	54.4^ab^	60.9^b^	52.3^ab^				
CH_4_/CO_2_ (ml L^−1^)	0	193	193	187	187	193	187	193	187	193	16.5	0.394	0.033	0.002
	150	192	189	200	192	220	258	200	208	195				
	300	190	181	207	185	197	192	196	227	197				
	500	189	180	240	207	193	170	179	190	190				

Note

Values are presented as mean and standard error of the mean (SEM). Numbers with different superscripts (a‐g) in a row differ significantly. Numbers with different superscripts (X‐Z) in a column differ significantly.

Abbreviations: AP, apple; AR, aronia; BR, beetroot; CA, carrot; OR, orange; PG, pomegranate; RG, red grape; TO, tomato; WG, white grape.

The comparison of pomaces among each other revealed more significant effects concerning the fermentation parameters. Total gas production, IVOMD, dOM and total gas per dOM were affected at both 300 and 500 g kg^−1^ dosages whereas methane and CO_2_ production were affected only by the 500 g kg^−1^ dosage. Total gas production (ml/g DM) and IVOMD were significantly lower in AR compared to AP and CA at both 300 and 500 g kg^−1^ concentration. The gas production with PG500 did not differ from that of AR, OR, RG, TO and WG at the same concentration. The methane production (ml g^−1^ DM) was similar between AR500 and most of the other treatments and significantly differed only from that of CA500. For PG500, the methane production was lowest and significantly differed from that of BR500 and CA500. Similarly, methane production per dOM (ml/g) was significantly lower in PG500 compared to TO500. No significant differences between pomaces were observed for any of the fermentation parameters tested at a concentration of 150 g kg^−1^.

For bacteria (2.05 ± 0.09 × 10^9^/ml incubation fluid) and protozoa (2.69 ± 0.19 × 10^4^/ml incubation fluid), no significant differences were observed due to treatment, concentration or their interaction after 24 h of incubation.

### 3.3 Production of short‐chain fatty acids

None of the pomaces had a significant effect on the formation of total or individual SCFA when compared to hay (Table 3). Most differences between pomaces occurred at 500 g kg^−1^ concentration and none occurred at 150 g kg^−1^ concentration. The production of total SCFA among the 500 g kg^−1^ dosages was lowest with AR and PG and significantly differed from that of AP, BR, CA, OR, TO and WG. The total SCFA production with RG500 was significantly lower compared to CA500 and OR500. The PG pomace significantly reduced total SCFA production compared to BR, CA and OR already among the 300 g kg^−1^ concentrations. Incubation with AR500, PG500 and RG500 significantly decreased acetate production compared to CA500 and BR500. The acetate production was also decreased with AR500 and PG500 (but not with RG500) compared to OR500 and TO500. In addition, AR500 resulted in a lower acetate production than the AP500 treatment. The acetate production with WG500 was only slightly higher compared to that of RG500 but it differed significantly only from that of CA500. The propionate production during PG500 incubation was lowest and significantly different from that of AP500, BR500, CA500, OR500 and WG500. Likewise, the PG500 resulted in the lowest butyrate production which significantly differed from that observed with CA500 and OR500. Within CA, the butyrate production was higher with CA500 than with CA300 which was the only concentration‐dependent effect observed for SCFA production. For valeric acid, no significant differences were observed. The ratio of acetate to propionate was also not significantly affected by any of the pomaces.

**Table 3:** Effect of fruit and vegetable pomaces on the *in vitro* production of short‐chain fatty acids (mmol/L) at dosages of 150, 300 and 500 g kg^−1^ of basal diet in comparison to a non‐supplemented control (*n*=6). Values are presented as mean and standard error of the mean (SEM)

	Dosage g kg^−1^	AP	AR	BR	CA	OR	PG	RG	TO	WG	SEM	*p*‐values		
												pomace	conc.	*p* × c
Acetate	0	30.6	30.6	31.8	31.8	30.6	31.8	30.6	31.8	30.6	2.52	<0.001	0.318	0.019
	150	31.1	30.0	33.5	33.4	34.4	29.7	28.9	32.4	29.3				
	300	31.3^ab^	28.1^ab^	34.8^b^	33.6^ab^	33.7^ab^	27.6^a^	30.0^ab^	32.9^ab^	29.7^ab^				
	500	32.5^bcde^	24.3^a^	37.6^de^	37.6^e^	36.5^cde^	26.6^ab^	28.7^abc^	34.9^cde^	30.6^abcd^				
Propionate	0	7.75	7.75	8.56	8.56	7.75	8.56	7.75	8.56	7.75	0.974	<0.001	0.033	<0.001
	150	8.65	8.25	8.28	8.40	9.08	7.18	8.16	8.18	8.19				
	300	9.01	7.65	8.64	8.30	9.51	6.07	8.34	8.13	8.77				
	500	9.48^b^	6.70^ab^	9.35^b^	9.83^b^	10.20^b^	5.61^a^	8.66^ab^	8.51^ab^	9.53^b^				
A:P ratio	0	3.94	3.94	3.83	3.83	3.94	3.83	3.94	3.83	3.94	0.461	<0.001	0.802	0.040
	150	3.67	3.73	4.11	4.10	3.86	4.24	3.68	4.02	3.67				
	300	3.56	3.79	4.10	4.11	3.57	4.60	3.73	4.10	3.51				
	500	3.64	3.73	4.06	3.90	3.70	4.79	3.45	4.15	3.31				
Butyrate	0	3.43	3.43	3.68	3.68^YZ^	3.43	3.68	3.43	3.68	3.43	0.641	<0.001	<0.001	0.370
	150	3.91	3.08	3.56	3.86^YZ^	4.13	3.15	3.71	3.75	3.81				
	300	4.13	2.97	3.81	4.44^Y^	4.40	2.87	3.92	3.81	3.78				
	500	4.87^ab^	2.72^ab^	4.63^ab^	5.16^bZ^	4.67^b^	3.22^a^	4.12^ab^	4.05^ab^	4.30^ab^				
Valerate	0	0.52	0.52	0.67	0.67	0.52	0.67	0.52	0.67	0.52	0.096	<0.001	0.045	0.818
	150	0.47	0.40	0.55	0.53	0.60	0.44	0.41	0.54	0.48				
	300	0.41	0.34	0.47	0.45	0.61	0.34	0.51	0.47	0.42				
	500	0.37	0.28	0.51	0.40	0.59	0.26	0.44	0.53	0.38				
Total SCFA	0	43.2	43.2	45.2	45.2	43.2	45.2	43.2	45.2	43.2	3.84	<0.001	0.192	0.007
	150	45.1	42.7	46.0	46.2	48.7	40.1	41.8	45.2	42.6				
	300	45.4^ab^	39.8^ab^	47.5^b^	46.6^b^	48.5^b^	33.8^a^	43.2^ab^	45.6^ab^	43.3^ab^				
	500	47.6^bc^	34.4^a^	51.7^bc^	52.9^c^	52.2^c^	34.8^a^	42.0^ab^	48.4^bc^	45.2^bc^				

Note

Numbers with different superscripts (a‐e) in a row differ significantly. Numbers with different superscripts (Y‐Z) in a column differ significantly.

Abbreviations: AP, apple; AR, aronia; BR, beetroot; CA, carrot; OR, orange; PG, pomegranate; RG, red grape; SCFA, short‐chain fatty acids; TO, tomato; WG, white grape.

## 4 DISCUSSION

In order to be considered a suitable feed for increased sustainability of ruminant nutrition, pomaces must not adversely affect ruminal fermentation processes which would compromise the energy supply to the animal. In the present study, the gas formation during fermentation, the amount of formed methane, the production of SCFA and the microbial counts were analysed to determine the effects of the pomaces on the fermentation intensity. Overall, only few effects were detected when comparing the pomaces to hay.

The pomaces applied in the present study differed largely in their polyphenol composition. For all vegetable pomaces (BR, CA, TO), only small proportions of NTP accompanied by hardly any tannins were found. This may in part explain why these pomaces, independent of the tested concentrations, hardly affected the *in vitro* fermentation characteristics compared to the basal diet (hay). In contrast to the lack of effects of TO shown in the present study, Marcos et al. (2019) observed an increased fermentation rate and SCFA production in vitro when incubating tomato pomace with a high‐concentrate diet. In line with our results, methane formation was not affected. Based on these findings and an IVOMD comparable to that of hay, TO can be considered as a suitable feed for ruminants. However, in a feeding experiment with sheep, Abbeddou et al. (2011) observed a slightly impaired palatability of feed containing tomato pomace accompanied by a reduction in milk yield and milk protein content while milk fat content was increased. The same study confirmed our observation that tomato pomace does not affect bacterial counts in rumen fluid.

The observed increase in total gas production per g DM by AP500 and CA500 indicates an increased fermentation activity. In case of AP500, this also increased the IVOMD. The apparent nutrient digestibility of OM of a diet containing 200 g kg^−1^ DM dried apple pomace determined in a study by Taasoli and Kafilzadeh (2008) in sheep was similar compared to that of a diet containing 150 and 300 g kg^−1^ dried AP determined in the present study (699 versus 665 and 686 g kg^−1^, respectively). When Mirzaei‐Aghsaghali et al. (2011) incubated pure dried apple pomace alone, the IVOMD was determined to be 715 g kg^−1^, which is comparable to the 728 g kg^−1^ IVOMD of AP500 in the present study.

The increased gas production in AP500 and CA500 may have been caused by their comparably higher non‐fibre carbohydrate content. The non‐fibre carbohydrates were not analysed in the present study but have been described earlier to be rather high in apple and carrot pomace (Pieszka et al., 2015; Sato et al., 2010). The increased methane formation observed with CA500 was likely caused by its 50% higher aNDFom proportion compared to hay. Consequently, such a high dosage of CA should be avoided in ruminant nutrition when aiming to reduce the greenhouse gas emissions. The inclusion of apple pomace into ruminant diets has been considered safe by previous studies (Wadhwa et al., 2015). Feeding a diet containing 200 g kg^−1^ dried apple pomace to finishing Sanjabi lambs even improved average daily gains and feed conversion ratio compared to the control diet containing alfalfa hay and barley grain (Taasoli & Kafilzadeh, 2008).

Studies on the effect of orange pomace on rumen fermentation are scarce as most studies investigate citrus pomace as a mix of different citrus fruits. However, feeding dry orange pulp to rumen‐cannulated ewes at a level of 500 g kg^−1^ of the diet did not affect organic matter digestibility, rumen pH and production of total SCFA (Barrios‐Urdanetat et al., 2003). These in vivo results confirm the results obtained with OR in the present in vitro study.

The inhibited gas production and decreased IVOMD with AR500 are in accordance with earlier reports on CT‐rich material. Since CT can bind to carbohydrates and proteins (from feed, but also microbial enzymes), their degradation in the rumen is inhibited (McSweeney et al., 2001). Monomers forming CT, such as the flavanols gallocatechin and epigallocatechin gallate, may decrease gas production and IVOMD in vitro, whereas epicatechin and quercetin did not influence these parameters in a previous study (Sinz et al., 2018). The AR polyphenols are mainly composed of cyanidin and polymeric proanthocyanidins (CT) and lower amounts of chlorogenic acids, quercetin and epicatechin (Määttä‐Riihinen et al., 2004; Pieszka et al., 2015). Considering the results of Sinz et al. (2018), the inhibition of rumen fermentation by AR can likely be ascribed to the CT and not to monomeric epicatechin or quercetin. Interestingly, the other two pomaces applied in the present study that contained rather high proportions of CT, precisely RG and WG, did not affect total gas production or IVOMD.

Despite similar TEP and TT proportions, AR and PG differently affected *in vitro* fermentation. In contrast to AR500, PG500 did not affect gas formation and IVOMD. This difference may in part be explained by the lower dOM content in AR500 compared to hay while the dOM content in PG500 did not differ from that of hay. The higher lignin (+92%) and lower CP (−50%) proportions in AR compared to PG likely contributed to the difference in dOM because aNDFom and ether extract proportions were more similar (30% and 20% difference, respectively). Another important factor contributing to this differing effect in fermentation parameters may be the considerably different composition of the TT portion, which, in PG, was clearly dominated by HT. This difference presumably contributed to the reduced digestibility observed in AR but not in PG and is in line with previous in vitro observations of a more pronounced reduction in gas production and digestibility with CT compared to HT (Makkar et al., 1995).

The differing tannin profiles are particularly intriguing as PG but not AR reduced the methane formation compared to hay. Former in vitro studies have shown methane mitigation potential of both CT and HT by either direct inhibition of methanogens or by an indirect effect via protozoa (Bhatta et al., 2009; Jayanegara et al., 2015). Even though most studies were performed with CT, the results regarding effects on methane formation are inconsistent (Aboagye & Beauchemin, 2019). This can be explained by a higher structural variability among the larger CT molecules compared to the smaller HT. In this context, it is worth noting that in the present study, all pomaces rich in CT but poor in HT (AR, RG, WG) did not affect methane formation. In contrast, both red and white grape pomace (containing more CT than HT) have been reported to reduce methane production in vitro with red grape pomace being more effective than white grape pomace (Russo et al., 2017). A recent study reported reduction of methane emission by 15% when dairy cows were fed pomace from either red or white grapes at a level of about 250 g kg^−1^ of DM, but this was accompanied by a 10% decrease in milk yield (Moate et al., 2020). To the best of our knowledge, dietary aronia pomace has not yet been evaluated regarding methane formation in ruminants.

There is only a limited number of studies applying HT in vivo but these show a quite consistent decrease in methane production (Aboagye et al., 2019; Stewart et al., 2019; Yang et al., 2016). It is suggested that this effect is mediated by gallic acid, a subunit of HT (Aboagye et al., 2019). Interestingly, even at a concentration of 500 g kg^−1^, PG did not reduce IVOMD which contradicts the in vitro results of Jayanegara et al. (2015) who reported a quadratic decrease in IVOMD with increasing concentrations of HT. Results comparable to the PG‐mediated methane mitigation observed in the present study were previously obtained with pomegranate extracts investigated in vitro and in vivo. In vitro, pomegranate extract (20 g kg^−1^ DM) resulted in a reduced methanogenesis (Singh et al., 2018). Supplementing buffaloes with pomegranate extract (20 g kg^−1^ of DM), rich in polyphenols (164 g kg^−1^ DM), resulted in decreased methane emissions which was accompanied by a higher weight gain compared to control at unchanged DM intake (Hundal et al., 2019). In line with these findings, improved digestibility of DM was observed in cows supplemented with pomegranate extract (Jami et al., 2012). Therefore, PG polyphenols seem to be effective in reducing methane without impairing palatability or feed digestibility and thus may improve ruminant productivity.

Even though microbial counts were not affected by PG in the present study, it can be assumed that shifts in the composition of the microbiome have occurred following shifts in the availability of different kinds of organic material as has been previously reported (Henderson et al., 2015). These shifts of microbial composition may have inhibited the metabolic activity of methanogens in case of the PG. Formation of propionate and methane production are competitive (Cieslak et al., 2012) but PG did not affect any of the SCFA in the present study. We therefore suggest that one or more pomegranate polyphenols, likely the HT or their metabolites, might modulate specific pathways involved in methanogenesis.

It must be considered that ingesting high concentrations of HT (e.g. >5 g/100 g DM) has resulted in severe adverse effects in ruminants (Garg et al., 1992; McSweeney et al., 1988). An inclusion of PG at 500 g kg^−1^ DM in the diet of ruminants would result in an additional TT supply of 56 g kg^−1^ DM with 50 g kg^−1^ DM consisting of HT which is quite close to the maximum recommendation (Kumar & Vaithiyanathan, 1990). In the present study, reduced methane formation was already observed at 150 g kg^−1^ proportion of PG compared to hay which can likely be considered a safe dosage, providing an additional HT supply of only 16 g kg^−1^ DM. In vivo testing will be an important step to further evaluate the applicability of PG as a potential methane mitigating feed compound for ruminants.

## 5 CONCLUSIONS

Since OR, RG and TO did not affect any of the fermentation parameters investigated in the present study, it can be assumed that these pomaces can be fed to ruminants in dietary proportions of up to 500 g kg^−1^. Pomaces of AR, BR, CA and WG seem suitable in proportions of up to 300 g kg^−1^ in ruminant diets. The present results only partly confirm the hypothesis (a) that low proportions of pomaces could be applied without changing rumen characteristics. At the same time, the results disprove hypothesis (b) as increasing pomace and consequently polyphenol contents did not necessarily affect the IVOMD. Differences in total gas and methane formation in AR and PG despite similarly high tannin proportions disprove hypothesis (c) that pomaces with a high tannin content reduce methane formation. The results emphasize a differing methane mitigation potential of the structurally different tannin subgroups. The lowered methane production at unchanged IVOMD with PG at a proportion as low as 150 g kg^−1^ of the diet makes this pomace an interesting feed component that deserves to be investigated in more detail as a potential agent to increase sustainability of ruminant nutrition not only by replacing fibre and sugar‐rich feed components but also by mitigating methane emission. This needs to be confirmed in in vivo studies. In addition, it must be considered that a prolonged incubation period of 48 h might have resulted in methane mitigation also by other pomaces. Therefore, a potential delayed methane mitigating effect would also be worth investigating.

## ANIMAL WELFARE STATEMENT

The authors confirm that the ethical policies of the journal, as noted on the journal's author guidelines page, have been adhered to and the appropriate ethical review committee approval has been received. The authors confirm that they have followed EU standards for the protection of animals used for scientific purposes and feed legislation.
